# Formulas for estimating the costs averted by sexually transmitted infection (STI) prevention programs in the United States

**DOI:** 10.1186/1478-7547-6-10

**Published:** 2008-05-23

**Authors:** Harrell W Chesson, Dayne Collins, Kathryn Koski

**Affiliations:** 1 Division of STD Prevention, National Center for HIV/AIDS, Viral Hepatitis, STD, and TB Prevention, Centers for Disease Control and Prevention, Atlanta, Georgia, USA

## Abstract

**Background:**

Sexually transmitted infection (STI) prevention programs can mitigate the health and economic burden of STIs. A tool to estimate the economic benefits of STI programs could prove useful to STI program personnel.

**Methods:**

We developed formulas that can be applied to estimate the direct medical costs and indirect costs (lost productivity) averted by STI programs in the United States. Costs and probabilities for these formulas were based primarily on published studies.

**Results:**

We present a series of formulas that can be used to estimate the economic benefits of STI prevention (in 2006 US dollars), using data routinely collected by STI programs. For example, the averted sequelae costs associated with treating women for chlamydia is given as (C_w_)(0.16)(0.925)(0.70)($1,995), where C_w _is the number of infected women treated for chlamydia, 0.16 is the absolute reduction in the probability of pelvic inflammatory disease (PID) as a result of treatment, 0.925 is an adjustment factor to prevent double-counting of PID averted in women with both chlamydia and gonorrhea, 0.70 is an adjustment factor to account for the possibility of re-infection, and $1,995 is the average cost per case of PID, based on published sources.

**Conclusion:**

The formulas developed in this study can be a useful tool for STI program personnel to generate evidence-based estimates of the economic impact of their program and can facilitate the assessment of the cost-effectiveness of their activities.

## Background

An estimated 19 million new cases of sexually transmitted infections (STIs) occur each year in the United States, with a price tag of $12 to $20 billion (including HIV) in lifetime direct medical costs (in 2006 US dollars) [1-5]. The indirect costs (such as lost productivity) associated with STIs are substantial as well. For example, the lifetime indirect cost per case of HIV in the US is almost $1 million [6].

STI prevention programs can mitigate the health and economic burden of STIs. A tool to estimate the economic benefits of STI programs could prove useful to STI program personnel. In 1992, the Centers for Disease Control and Prevention (CDC) provided a series of formulas that program personnel could apply to estimate the medical costs offset by the prevention activities of their program [7]. Since that time, the costs of STIs have changed substantially, rendering the 1992 CDC model somewhat dated. To address the need for more current tools, we developed formulas (loosely based on the 1992 CDC model) that can be applied to estimate the direct medical costs and indirect costs (lost productivity) averted by STI programs in the US.

## Methods

We applied a societal perspective and included all relevant costs regardless of who pays these costs [8,9]. We developed formulas that can be applied to estimate the direct medical costs and indirect costs (lost productivity) averted by STI programs. We focused on the benefits of treating people with primary and secondary (P&S) syphilis, gonorrhea, and chlamydia, and the benefits of HIV counseling and testing. These benefits included the sequelae costs averted by treatment of people with STIs, the prevention of congenital syphilis in infants born to mothers treated for P&S syphilis, the interruption of STI transmission in the population, the reduction in STI-attributable HIV infections (HIV infections that would not have occurred without the facilitative effects of STIs on HIV transmission and acquisition), HIV infections averted by HIV counseling and testing, and the corresponding reductions in lost productivity. Costs and probabilities for these formulas were based on published studies and assumptions, as listed in Table 1 and described below. The first five sections below focus on direct medical costs, and the final section examines indirect costs (lost productivity). Costs were adjusted for inflation to year 2006 US dollars using the medical care component and the all items component (for direct medical costs and indirect costs, respectively) of the consumer price index for all urban consumers from the US Department of Labor, Bureau of Labor Statistics.

**Table 1:** Summary of STI cost estimates (in 2006 US dollars) and selected parameter values applied in the formulas

**Parameter**	**Value applied**		**Range applied**
**Direct medical costs**	**Females**	**Males**	
Average cost per case of PID [23–25]	$1,995	not applicable	± 50%
Average cost per case of epididymitis [26]	not applicable	$274	± 50%
Average sequelae costs per case of syphilis [5]	$572*	$572*	± 50%
Average cost per case of chlamydia [5]	$315	$26	± 50%
Average cost per case of gonorrhea [5]	$343	$68	± 50%
Average cost per case of syphilis [5]	$572*	$572*	± 50%
Average cost per case of HIV [6]	$198,471	$198,471	± 50%
Average cost per case of congenital syphilis [1,64,65]	$6,738	$6,738	± 50%
**Indirect (lost productivity) costs**			
Average cost per case of HIV [6]	$831,614	$831,614	± 50%
Average cost per untreated case of chlamydia [85]	$148	$13	± 50%
Average cost per untreated case of gonorrhea**	$171	$34	± 50%
Average cost per untreated case of syphilis**	$112*	$112*	± 50%
Average cost per case of chlamydia**	$47	$10	± 50%
Average cost per case of gonorrhea**	$47	$10	± 50%
Average cost per case of syphilis**	$112*	$112*	± 50%
Average cost per case of congenital syphilis**	$60,421	$60,421	± 50%
**Other parameters**			
Absolute reduction in probability of sequelae due to treatment: chlamydia**	0.16	0.03	± 90%
Absolute reduction in probability of sequelae due to treatment: gonorrhea**	0.14	0.03	± 90%
Adjustment to chlamydia costs averted to account for gonorrhea coinfection**	0.925	0.925	± 5%
Adjustment to gonorrhea costs averted to account for chlamydia coinfection**	0.79	0.90	± 5%
Adjustment to account for reinfection: gonorrhea and chlamydia**	0.70	0.70	± 25%
Probability of congenital syphilis given untreated syphilis in mother [63]	0.50	not applicable	± 50%
Number of cases of STI averted in population per STI case treated**	0.50	0.50	± 90%
Probability of a new case of HIV attributable to chlamydia [70]	0.0011	0.0011	± 90%
Probability of a new case of HIV attributable to gonorrhea [70]	0.0007	0.0007	± 90%
Probability of a new case of HIV attributable to syphilis [70]	0.02386	0.02386	± 90%
Adjustment for time frame for STI-attributable HIV infections**	0.25	0.25	± 90%
Adjustment for partner overlap (heterosexuals) [67]	0.75	0.75	± 25%
Adjustment for partner overlap (MSM)**	not applicable	0.50	± 25%
Additional adjustment for averted HIV costs for MSM**	not applicable	0.25	not varied
HIV cases averted per person counseled and tested [78,81]	0.00045	0.00045	± 90%
Adjustment for repeat counseling and testing**	0.875	0.875	± 10%

*The average sequelae cost per case of syphilis was set equal to the average cost per case of syphilis (and the indirect cost per case of syphilis was set equal to the indirect cost per case of untreated syphilis), because when calculating the costs of syphilis we allowed for the possibility that treatment of syphilis would have occurred (even in the absence of the STI program) before the onset of sequelae.

**See text for sources, assumptions, and additional information.

### Sequelae costs averted by treatment of people with chlamydia, gonorrhea, and P&S syphilis

Formulas for estimating the sequelae costs averted by treatment of chlamydia and gonorrhea were based on published estimates of the impact of STI treatment on the probability of developing pelvic inflammatory disease (PID) in women or epididymitis in men and the average costs per case of PID and epididymitis. We assumed the probability of PID in women would be reduced from 20% to 4% by treatment of chlamydia [5,10-18] and would be reduced from 20% to 6% by treatment of gonorrhea [5,10,11,13-16,19,20]. We assumed the probability of epididymitis in men would be reduced from 3% to 0% by treatment of gonorrhea or chlamydia [5,10,21,22]. We applied $1,995 as the direct medical cost per case of PID (the average of three published estimates, $1,621 [23], $2,772 [24], and $1,592 [25]), which includes the costs of care for acute PID and costs associated with sequelae such as chronic pelvic pain, ectopic pregnancy, and infertility. We applied $274 as the direct medical cost per case of epididymitis [26].

For P&S syphilis in men and women, the average cost averted per case treated we applied was $572 [5]. This cost includes the possibility of neurosyphilis and cardiovascular syphilis in untreated syphilis cases, and allows for the possibility that treatment of syphilis would have occurred subsequently (either by the infected person seeking treatment or receiving treatment inadvertently through administration of antibiotics for an unrelated health condition), before the advent of sequelae [5]. We included the possibility of subsequent treatment before the advent of sequelae for syphilis (but not for gonorrhea and chlamydia) because the time span from infection to sequelae can be substantially longer for syphilis than for gonorrhea and chlamydia [5,23].

The number of infected people treated was calculated as the number of treated people with laboratory-confirmed infections, plus Q times the number of treated people with clinical diagnosis of infection, plus R times the number of people treated presumptively because of sexual contact with a partner known or suspected to be infected, where Q and R are defined as follows. Q is the probability that a person with a clinical diagnosis of a given STI is actually infected with that STI. We applied values of Q of 20% for chlamydia and gonorrhea in women, 35% for chlamydia and gonorrhea in men, and 70% for syphilis in men and women, based on published reports of the utility of syndromic diagnoses and the frequency of chlamydia as a cause of male nongonococcal urethritis [27-33]. R is the probability that the sex partner of an infected person is also infected. We applied values of R of 57% for chlamydia and 46% for gonorrhea, based on studies of partner notification [34-36]. We applied a value of R of 30% for syphilis, based on studies of partner notification [35,37-39] as well as estimates of the per-partnership transmission probability of syphilis [40,41].

We also allowed the possibility that people with gonorrhea might be treated presumptively for chlamydia, and vice-versa. To incorporate this possibility, we assumed that 20% and 42% of men and women, respectively, with gonorrhea who were treated presumptively for chlamydia did indeed have chlamydia, based on a study of coinfection in STI clinic attendees in the US [42] and consistent with coinfection studies in other settings [43-49]. We assumed that 15% of men and women with chlamydia who were treated presumptively for gonorrhea did indeed have gonorrhea [42-48].

Regardless of the reason for treatment (laboratory-confirmed diagnosis, clinical diagnosis, partner services, or presumptive treatment for dual infection) for gonorrhea and chlamydia, we reduced the estimated impact of treatment by multiplying by two adjustment factors. The first adjustment factor (0.925 for men and women treated for chlamydia, and 0.79 and 0.9 for women and men, respectively, treated for gonorrhea) was based on the probability of gonorrhea and chlamydia coinfection described above and was included to mitigate potential overestimation of the benefits of preventing PID or epididymitis in people with both gonorrhea and chlamydia. The second adjustment factor (0.70 for men and women treated for gonorrhea, chlamydia, or both) was included to account for the possibility of re-infection within one year of treatment [50-62], which could offset (at least partially) the benefits of treatment.

Estimates of the number of treated partners may be unavailable in the event of patient-delivered partner therapy. In such cases, a reasonable approximation is that, on average, one partner is treated for each patient provided with therapy for his or her partner(s) [53]. This average reflects the possibility that some patients might give the medication to none, one, or more than one of their partners.

### Congenital syphilis treatment costs averted by treatment of P&S syphilis in women

We assumed that in the absence of treatment, 50% of pregnant women with P&S syphilis would have delivered a child with congenital syphilis [63]. The first-year direct medical cost estimate we applied for congenital syphilis was $6,738 [1,64,65]. The estimated averted costs are likely understated because we did not assign a cost to premature births and stillbirths, or costs of congenital syphilis beyond one year.

### Treatment and sequelae costs averted by reducing transmission of chlamydia, gonorrhea, and syphilis in the population

We assumed that each STI case treated prevents, on average, 0.5 cases of that STI in the population by interrupting the transmission of that STI. This assumption is based in part on a model-based evaluation of chlamydia screening, in which the estimated number of adverse outcomes prevented when the population-level benefits of screening were addressed was roughly double the estimated number of adverse outcomes prevented when population-level benefits were not modeled [66]. These modeling results are consistent with an assumption that each STI case treated would prevent, on average, one additional case of that STI in the population. However, to account for possible overlap in the sex partners of people treated [67] and the possibility that secondary transmission(s) from the infected person had already occurred prior to treatment, we reduced the expected population-level impact by 50%, thereby assuming that each case of STI treated prevents 0.5 cases (rather than one case) of that STI in the population.

To calculate the treatment and sequelae costs averted by the interruption of STI transmission, we applied published estimates of the average lifetime cost per case of syphilis, gonorrhea, and chlamydia, as these estimates incorporate the probability and cost of STI treatment as well as the probability and cost of adverse sequelae in the absence of treatment. The estimated average lifetime direct medical costs per case we applied were: $315 and $26 for chlamydia in women and men, respectively; $343 and $68 for gonorrhea in women and men, respectively, and $572 for syphilis in men and women [5]. The average treatment and sequelae cost per case of syphilis we applied ($572) was the same value we applied above for the sequelae cost averted per case of syphilis treated, because when calculating the sequelae cost of syphilis averted by treatment we allowed for the possibility of subsequent treatment for syphilis before the advent of sequelae.

In assessing the costs averted by the interruption of STI transmission by treatment of STIs in heterosexuals, we applied the average cost per case of that STI in women and men ($171 for chlamydia, $206 for gonorrhea, and $572 for syphilis), because treatment of a person with a given STI would be expected to reduce treatment and sequelae costs not only in his or her opposite-sex partners, but in the partners' subsequent opposite-sex partners as well, and so on. In assessing the costs averted by the interruption of STI transmission by treatment of STIs in men who have sex with men (MSM), we applied the STI costs per case in men.

In developing the formula for costs averted through the interruption of STI transmission, we excluded people treated for STIs as a result of partner notification, to reduce potential double-counting of the benefits of preventing STIs in partners of infected people treated for STIs.

### HIV costs averted by reducing HIV transmission through treatment of chlamydia, gonorrhea, and P&S syphilis

Because STIs can facilitate the acquisition and transmission of HIV [68], treatment of STIs can reduce the incidence of HIV [69]. Thus, treatment of STIs offer the additional economic benefit of reducing HIV costs as well [70].

The average number of HIV cases attributable to each new case of chlamydia, gonorrhea, and P&S syphilis in heterosexuals has been estimated at 0.0011, 0.0007, and 0.02386, respectively [70]. These estimates are based on the facilitative effects of the STI on HIV transmission and acquisition from the time of acquisition of the STI. We assumed that the treatment of the STI reduces the time frame in which an STI-attributable HIV transmission is possible by one-fourth. That is, in terms of preventing STI-attributable HIV cases, we assumed that treating an STI provided only one-fourth the potential benefit of preventing the STI altogether. Thus, the above-listed probabilities were multiplied by 0.25 in our application. The resulting estimate was then multiplied by 0.75 to account for overlap in sex partners [67] of people treated by a given STI program.

For the expected number of STI-attributable HIV infections per case of STI in MSM, we applied the same estimates as above for heterosexuals, except that we applied an adjustment factor of 0.50 (rather than 0.75) to account for partner overlap, owing to higher numbers of casual and anonymous partners in MSM at high risk for STIs and HIV than in heterosexual men [37,71-75]. We applied an additional adjustment factor of 0.25 for MSM because, in populations at high risk of acquiring HIV, a substantial proportion of the estimated HIV cases prevented may actually be "delayed" rather than "forever averted" by prevention efforts [76], and to account for "HIV sero-sorting" in which partners are selected based on HIV status [77].

In developing the formula for costs averted by preventing STI-attributable HIV infections, we excluded people treated for STIs as a result of partner notification, to reduce potential double-counting of the benefits of preventing STI-attributable HIV infections in partners of infected people treated for STIs.

We applied a lifetime direct medical cost per case of HIV of $198,471 for both men and women [6].

### HIV costs averted by HIV counseling and testing

HIV counseling and testing can reduce HIV incidence by reducing not only the probability that a person with HIV will transmit the virus (through behavioral changes due to counseling and virologic effects of antiretroviral therapy), but also the probability that a person without HIV will become infected [78-80]. One published decision analysis model suggested that HIV counseling and testing, when provided to a cohort of 10,000 people with 1.5% HIV seroprevalence, would avert 8 cases of HIV [78]. Another published model suggested that roughly 1 case of HIV would be prevented per 10,000 people screened [81]. We applied the average of these two estimates, thereby assuming that an expected 0.00045 cases of HIV are averted for each person counseled and tested. As described above, we applied an adjustment to account for partner overlap (0.75 for heterosexuals and 0.5 for MSM), and a further adjustment factor (0.25) for MSM to account for sero-sorting in the absence of counseling and testing and for the possibility that HIV infections prevented are not forever averted but merely delayed. We also applied an additional adjustment factor (0.875) to mitigate the double-counting of benefits in people seeking repeat counseling and testing [82,83].

As the incidence of HIV in populations served by counseling and testing programs can exceed 1% annually [84], only modest reductions in HIV risk behaviors would be needed to achieve the per-person reduction in HIV incidence we applied in this exercise.

### Indirect costs (lost productivity) averted

Our estimates of the indirect costs of STIs focused on lost productivity. The lost productivity per case of HIV has been estimated at $831,614 [6]. The lost productivity per case of untreated chlamydia in females has been estimated at $148 [85]. To our knowledge, estimates of the lost productivity associated with untreated STIs were not available for chlamydia in males, and for gonorrhea and syphilis in males and females at the time this study was conducted. For these STIs, we assumed that the ratio of indirect costs per untreated case to lifetime direct medical costs per case was 0.5, roughly the same as for chlamydia in females ($148/$315). The use of such ratios to estimate indirect costs is based on the assumption that indirect and direct costs of a given disease are usually related to the severity of the disease. Ratios of indirect to direct costs consistent with our assumption of 0.5 have been applied elsewhere in other studies of the burden of STIs [86].

Using this 0.5 ratio, the estimated lost productivity per case of untreated STI was as follows: $13 for chlamydia in men; $171 and $34 for gonorrhea in women and men, respectively; and $286 for syphilis in men and women. The indirect cost for congenital syphilis using this formula is $3,369. However, to incorporate potentially lifelong impacts of congenital syphilis, we assumed this indirect cost of $3,369 was incurred every year for 25 years, for a total indirect cost of $60,421 (when applying a 3% annual discount rate).

The indirect costs estimates listed above for chlamydia, gonorrhea, and syphilis reflect the average cost per untreated case. For the purposes of this exercise, we also needed estimates of the average cost per case of chlamydia, gonorrhea, and syphilis that incorporate the probability of receiving treatment and avoiding sequelae-related indirect costs. To calculate estimates of the average indirect costs per case of STI, we applied the following probabilities of receiving treatment before the possible onset of sequelae: 68% and 22% for women and men, respectively, with chlamydia; 73% and 71% for women and men, respectively, with gonorrhea, and 61% for men and women with syphilis [5]. We conservatively assumed that treatment for STIs before the onset of sequelae imposed no indirect costs. Under these assumptions, the estimated indirect costs per case of STI were approximately as follows: $47 for chlamydia and gonorrhea in women, $10 for chlamydia and gonorrhea in men, and $112 for syphilis in men and women. In keeping with our assumption applied earlier that subsequent treatment of syphilis might occur before the onset of sequelae, we applied the same value ($112) for the indirect cost per case of syphilis and the indirect cost per untreated case of syphilis (Table 1).

We applied the indirect costs per case of STI for cases averted by the interruption of STI transmission in the population. For partners of heterosexuals, we applied the average indirect cost per case averted in men and women ($29 for chlamydia and gonorrhea, reflecting the average indirect cost per case of $47 in women and $10 in men, and $112 for syphilis). For partners of MSM, we applied the indirect cost per case in men ($10 for chlamydia and gonorrhea, and $112 for syphilis).

In calculating the indirect costs averted by treating people with STIs, we applied the estimated indirect cost per untreated case of STI.

### Sensitivity analyses

To address the uncertainty in the cost per case estimates and other parameter values, we applied a range of values as indicated in Table 1. We used Monte Carlo simulations [87] to generate a range of the most plausible estimates of the costs averted by STI prevention. We performed 50,000 simulations, each time drawing a random value for each parameter, assuming a triangular distribution between the parameter's lower and upper bound values. For each simulation, we calculated the relative change in the direct costs averted (the percentage difference between the averted direct costs in the simulation and the averted direct costs in the base case). For each simulation, we also calculated the relative change in the indirect costs averted, which for simplicity we calculated as the average of the relative change in indirect costs averted in treated people and the relative change in indirect costs averted in partners of treated people. We then used the 10^th ^and 90^th ^percentiles of these 50,000 simulations as the lower and upper bound values of the STI costs averted by STI program activities.

### Examples of averted cost calculations

To illustrate the use of the formulas, we examined the estimated costs averted by the treatment of 1,000 people with chlamydia, 500 people with gonorrhea, and 100 people with syphilis, assuming that everyone treated had a laboratory-confirmed infection. We also estimated the costs averted by HIV counseling and testing of 2,000 people. In all of these examples, we assumed that 60% of those served were men, and that 67% of the men were heterosexual.

## Results

The input needed from the STI program in order to apply the averted cost formulas is summarized in Table 2. The formulas to estimate the numbers of infected people treated for chlamydia, gonorrhea, and P&S syphilis are summarized in Table 3. The formulas used to estimate the averted costs (in 2006 US dollars) are presented in Tables 4, 5, 6.

**Table 2:** Summary of STI program data needed to apply the averted cost formulas

	All women	Heterosexual men	Men who have sex with men
Number of people treated: lab-confirmed infection			
Chlamydia	X_1_	Y_1_	Z_1_
Gonorrhea	X_2_	Y_2_	Z_2_
Syphilis	X_3_	Y_3_	Z_3_
Number of people treated: clinical diagnosis			
Chlamydia	X_4_	Y_4_	Z_4_
Gonorrhea	X_5_	Y_5_	Z_5_
Syphilis	X_6_	Y_6_	Z_6_
Number of partners treated*			
Chlamydia	X_7_	Y_7_	Z_7_
Gonorrhea	X_8_	Y_8_	Z_8_
Syphilis	X_9_	Y_9_	Z_9_
Number treated presumptively for chlamydia, based on gonorrhea diagnosis	X_10_	Y_10_	Z_10_
Number treated presumptively for gonorrhea, based on chlamydia diagnosis	X_11_	Y_11_	Z_11_
Number of people receiving HIV counseling and testing	X_12_	Y_12_	Z_12_
Number of pregnant women treated for syphilis, lab-confirmed diagnosis	X_13_	not applicable	not applicable
Number of pregnant women treated for syphilis, clinical diagnosis	X_14_	not applicable	not applicable
Number of pregnant women treated for syphilis, partner notification	X_15_	not applicable	not applicable

*Refers to those treated because of sexual contact with an infected person. For example, X_7 _refers to the number of women treated for chlamydia because of sexual contact with an infected person. Syphilis cases include primary and secondary (P&S) syphilis only.

**Table 3:** Summary of the estimated numbers of infected people treated for sexually transmitted infections

Symbol	Description	Formula
C_w_	Number of women with chlamydia treated	X_1 _+ 0.2(X_4_) + 0.57(X_7_) + 0.42(X_10_)
Ĉ_w_	Number of women with chlamydia treated, excluding partner services	X_1 _+ 0.2(X_4_) + 0.42(X_10_)
C_m_	Number of heterosexual men with chlamydia treated	Y_1 _+ 0.35(Y_4_) + 0.57(Y_7_) + 0.2(Y_10_)
Ĉ_m_	Number of heterosexual men with chlamydia treated, excluding partner services	Y_1 _+ 0.35(Y_4_) + 0.2(Y_10_)
C_msm_	Number of MSM with chlamydia treated	Z_1 _+ 0.35(Z_4_) + 0.57(Z_7_) + 0.2(Z_10_)
Ĉ_msm_	Number of MSM with chlamydia treated, excluding partner services	Z_1 _+ 0.35(Z_4_) + 0.2(Z_10_)
G_w_	Number of women with gonorrhea treated	X_2 _+ 0.2(X_5_) + 0.46(X_8_) + 0.15(X_11_)
Ĝ_w_	Number of women with gonorrhea treated, excluding partner services	X_2 _+ 0.2(X_5_) + 0.15(X_11_)
G_m_	Number of heterosexual men with gonorrhea treated	Y_2 _+ 0.35(Y_5_) + 0.46(Y_8_) + 0.15(Y_11_)
Ĝ_m_	Number of heterosexual men with gonorrhea treated, excluding partner services	Y_2 _+ 0.35(Y_5_) + 0.15(Y_11_)
G_msm_	Number of MSM with gonorrhea treated	Z_2 _+ 0.35(Z_5_) + 0.46(Z_8_) + 0.15(Z_11_)
Ĝ_msm_	Number of MSM with gonorrhea treated, excluding partner services	Z_2 _+ 0.35(Z_5_) + 0.15(Z_11_)
S_w_	Number of women with syphilis treated	X_3 _+ 0.7(X_6_) + 0.3(X_9_)
Ŝ_w_	Number of women with syphilis treated, excluding partner services	X_3 _+ 0.7(X_6_)
S_m_	Number of heterosexual men with syphilis treated	Y_3 _+ 0.7(Y_6_) + 0.3(Y_9_)
Ŝ_m_	Number of heterosexual men with syphilis treated, excluding partner services	Y_3 _+ 0.7(Y_6_)
S_msm_	Number of MSM with syphilis treated	Z_3 _+ 0.7(Z_6_) + 0.3(Z_9_)
Ŝ_msm_	Number of MSM with syphilis treated, excluding partner services	Z_3 _+ 0.7(Z_6_)

The X_i_, Y_i_, and Z_i _terms are defined in Table 2. Syphilis cases include primary and secondary (P&S) syphilis only.

**Table 4:** Formulas for estimating averted direct medical costs of chlamydia, gonorrhea, syphilis, and congenital syphilis

**Sequelae costs averted by treatment of people with chlamydia, gonorrhea, and syphilis**	
Chlamydia	[(C_w_)(0.16)(0.925)(0.70)($1,995)] + [(C_m_+C_msm_)(0.03)(0.925)(0.70)($274)]
Gonorrhea	[(G_w_)(0.14)(0.79)(0.70)($1,995)] + [(G_m_+G_msm_)(0.03)(0.90)(0.70)($274)]
Syphilis	(S_w_+S_m_+S_msm_)($572)
**Congenital syphilis treatment costs averted by treatment of syphilis in women**	
	[X_13 _+ 0.7(X_14_) + 0.3(X_15_)] [(0.5)($6,738)]
**Treatment and sequelae costs averted by reducing transmission of chlamydia, gonorrhea, and syphilis in the population**	
Chlamydia	[(Ĉ_w_+ Ĉ_m_)(0.5)($171)] + [(Ĉ_msm_)(0.5)($26)]
Gonorrhea	[(Ĝ_w_+ Ĝ_m_)(0.5)($206)] + [(Ĝ_msm_)(0.5)($68)]
Syphilis	(Ŝ_w_+Ŝ_m_+Ŝ_msm_)(0.5)($572)

The C_i_, G_i_, S_i_, Ĉ_i_, Ĝ_i_, and Ŝ_i _terms are defined in Table 3. The X_i_, Y_i_, and Z_i _terms are defined in Table 2. Syphilis cases include primary and secondary (P&S) syphilis only.

**Table 5:** Formulas for estimating averted direct medical costs of HIV

**HIV costs averted by reducing HIV transmission through treatment of chlamydia, gonorrhea, and syphilis**	
Chlamydia	[(Ĉ_w _+ Ĉ_m_)(0.0011)(0.25)(0.75)($198,471)] + [(Ĉ_msm_)(0.0011)(0.25)(0.50)(0.25)($198,471)]
Gonorrhea	[(Ĝ_w _+ Ĝ_m_)(0.0007)(0.25)(0.75)($198,471)] + [(Ĝ_msm_)(0.0007)(0.25)(0.50)(0.25)($198,471)]
Syphilis	[(Ŝ_w _+ Ŝ_m_)(0.02386)(0.25)(0.75)($198,471)] + [(Ŝ_msm_)(0.02386)(0.25)(0.50)(0.25)($198,471)]
**HIV costs averted by HIV counseling and testing**	
	[(X_12 _+ Y_12_)(0.00045)(0.75)(0.875)($198,471)] + [(Z_12_)(0.00045)(0.50)(0.25)(0.875)($198,471)]

The C_i_, G_i_, S_i_, Ĉ_i_, Ĝ_i_, and Ŝ_i _terms are defined in Table 3. The X_i_, Y_i_, and Z_i _terms are defined in Table 2. Syphilis cases include primary and secondary (P&S) syphilis only.

**Table 6:** Formulas for estimating averted indirect costs (lost productivity) of chlamydia, gonorrhea, syphilis, congenital syphilis, and HIV

**Indirect STI costs averted**	
Chlamydia	[(C_w_)(0.925)(0.70)($148)] + [(C_m_+ C_msm_)(0.925)(0.70)($13)] + [(Ĉ_w _+ Ĉ_m_)(0.5)($29)] + [(Ĉ_msm_)(0.5)($10)]
Gonorrhea	[(G_w_)(0.79)(0.70)($171)] + [(G_m_+ G_msm_)(0.9)(0.70)($34)] + [(Ĝ_w _+ Ĝ_m_)(0.5)($29)] + [(Ĝ_msm_)(0.5)($10)]
Syphilis	[(S_w_+S_m_+S_msm_)($112)] + [(Ŝ_w_+Ŝ_m_+Ŝ_msm_)(0.5)($112)]
Congenital syphilis	[X_13 _+ 0.7(X_14_) + 0.3(X_15_)] [(0.5)($60,421)]
**Indirect HIV costs averted by reducing HIV transmission through treatment of STIs**	
Chlamydia	[(Ĉ_w _+ Ĉ_m_)(0.0011)(0.25)(0.75)($831,614)] + [(Ĉ_msm_)(0.0011)(0.25)(0.5)(0.25)($831,614)]
Gonorrhea	[(Ĝ_w _+ Ĝ_m_)(0.0007)(0.25)(0.75)($831,614)] + [(Ĝ_msm_)(0.0007)(0.25)(0.5)(0.25)($831,614)]
Syphilis	[(Ŝ_w _+ Ŝ_m_)(0.02386)(0.25)(0.75)($831,614)] + [(Ŝ_msm_)(0.02386)(0.25)(0.5)(0.25)($831,614)]
**Indirect HIV costs averted by reducing HIV transmission through HIV counseling and testing**	
	[(X_12 _+ Y_12_)(0.00045)(0.75)(0.875)($831,614)] + [(Z_12_)(0.00045)(0.50)(0.25)(0.875)($831,614)]

The C_i_, G_i_, S_i_, Ĉ_i_, Ĝ_i_, and Ŝ_i _terms are defined in Table 3. The X_i_, Y_i_, and Z_i _terms are defined in Table 2. Syphilis cases include primary and secondary (P&S) syphilis only.

### Sequelae costs averted by treatment of people with chlamydia, gonorrhea, and P&S syphilis (Table 4, top)

For chlamydia, the formula includes the absolute reduction in the probability of sequelae associated with treatment (0.16 for women and 0.03 for men), the sequelae cost ($1,995 for women and $274 for men), an adjustment (0.925) to prevent double-counting of benefits of treating people with both gonorrhea and chlamydia, and an adjustment (0.70) to account for the possibility of re-infection. For gonorrhea, the formula includes the absolute reduction in the probability of sequelae associated with treatment (0.14 for women and 0.03 for men), the sequelae cost ($1,995 for women and $274 for men), an adjustment (0.79 for women and 0.9 for men) to prevent double-counting of benefits of treating people with both gonorrhea and chlamydia, and an adjustment (0.70) to account for the possibility of re-infection. For syphilis, the formula includes the cost per case of syphilis ($572).

### Congenital syphilis treatment costs averted by treatment of P&S syphilis in women (Table 4, middle)

These formulas include the terms 0.7 and 0.3 to represent the probability that women in the specific categories actually have syphilis. The term 0.5 reflects the probability of congenital syphilis in the absence of treatment, and the term $6,738 represents the direct medical cost of congenital syphilis.

### Treatment and sequelae costs averted by reducing transmission of chlamydia, gonorrhea, and syphilis in the population (Table 4, bottom)

In these formulas, the term 0.5 represents the number of cases of STI averted in the population per person treated for that STI. The average lifetime cost per case of STI is given by the terms $171 and $26 (for chlamydia in partners of heterosexuals and MSM, respectively) and $206 and $68 (for gonorrhea in partners of heterosexuals and MSM,, respectively), and $572 (for syphilis).

### HIV costs averted by reducing HIV transmission through treatment of chlamydia, gonorrhea, and P&S syphilis (Table 5, top)

These formulas include the probability that an STI-attributable HIV infection will occur per new case of STI (0.0011 for chlamydia, 0.0007 for gonorrhea, and 0.02386 for syphilis), an adjustment (0.25) reflecting the assumption that (in terms of preventing STI-attributable HIV infections) treating an STI provides only one-fourth the benefit of preventing the STI altogether, an adjustment to account for partner overlap (0.75 for heterosexuals and 0.5 for MSM), a further adjustment (0.25) for MSM to account for sero-sorting and for the possibility that HIV infections prevented are not forever averted but merely delayed, and the cost per case of HIV ($198,471).

### HIV costs averted by HIV counseling and testing (Table 5, bottom)

These formulas include the reduction in the probability of acquiring or transmitting HIV (0.00045), adjustment factors to account for partner overlap (0.75 for heterosexuals and 0.5 for MSM), a further adjustment for MSM (0.25) as described above, an adjustment to mitigate the double-counting of benefits in people seeking repeat counseling and testing (0.875), and the cost per case of HIV ($198,471).

### Indirect costs (lost productivity) averted (Table 6)

The formulas for the indirect STI costs averted include two main components. First, there are the benefits of treating people for STIs, calculated using the indirect cost per untreated case ($148 and $13 for chlamydia in women and men, $171 and $34 for gonorrhea in women and men, and $112 for syphilis in women and men). The adjustment terms 0.925 (for chlamydia), 0.79 and 0.90 (for gonorrhea in women and men, respectively) are applied to prevent double-counting of benefits of treating people with both gonorrhea and chlamydia. The adjustment term (0.70) accounts for the possibility of re-infection, which would reduce the benefits of treatment. Second, there are the benefits of preventing STIs in the population, calculated using the indirect cost per case estimates ($29 for chlamydia and gonorrhea averted in partners of heterosexuals, $10 for chlamydia and gonorrhea averted in partners of MSM, and $112 for syphilis in partners of heterosexuals and MSM). The 0.5 term is applied to reflect the expected number of STI infections averted in the population per person treated for a given STI.

The indirect cost formulas for congenital syphilis and HIV are the same as for the direct costs for these two items, except that the estimated indirect cost per case estimates ($60,421 for congenital syphilis and $831,614 for HIV) are applied rather than the direct cost estimates.

### Sensitivity analyses (Table 7)

The ranges of estimates for the costs averted by STI programs are shown in Table 7 as a function of the base case results. These ranges show the estimated 10^th ^and 90^th ^percentile of averted cost estimates that would result in 50,000 simulations in which the inputs in Table 1 were simultaneously varied between their lower and upper bounds (assuming a triangular distribution). Sequelae costs averted by treatment of people with chlamydia and gonorrhea varied substantially, owing primarily to uncertainty in the probability of sequelae in the absence of treatment. Treatment and sequelae costs averted by reducing STIs in the population varied substantially as well, given the uncertainty in estimating the population-level benefits of STI treatment of individuals. HIV costs averted varied more than any other category of costs, due in part to the uncertainty in the probability of averting a new case of HIV.

**Table 7:** Ranges of estimates of costs averted by STI programs: sensitivity analyses

**Cost estimated**	**Upper and lower bounds obtained in simulations (10^th ^and 90^th ^percentiles)**
Sequelae costs averted by treatment of people with chlamydia and gonorrhea	Base case - 54%, Base case + 60%
Sequelae costs averted by treatment of people with syphilis	Base case - 28%, Base case + 28%
Congenital syphilis treatment costs averted	Base case - 36%, Base case + 39%
Treatment and sequelae costs averted by reducing STIs in the population	Base case - 53%, Base case + 58%
HIV costs averted through treatment of STIs	Base case - 67%, Base case + 80%
HIV costs averted by HIV counseling and testing	Base case - 54%, Base case + 60%
Indirect chlamydia and gonorrhea costs averted	Base case - 35%, Base case + 38%
Indirect syphilis costs averted	Base case - 35%, Base case + 38%
Indirect congenital syphilis costs averted	Base case - 36%, Base case + 39%
Indirect HIV costs averted through treatment of STIs	Base case - 67%, Base case + 80%
Indirect HIV costs averted by HIV counseling and testing	Base case - 54%, Base case + 60%

### Examples of averted cost calculations (Table 8)

**Table 8:** Examples of estimated costs averted by STI program activities

**Costs averted**	Chlamydia treatment (1,000 people)	Gonorrhea treatment (500 people)	Syphilis treatment (100 people)	HIV C&T (2,000 people)
**Direct costs averted**				
STI sequelae costs in treated people	$85,870	$32,440	$57,200	$0
Congenital syphilis costs	$0	$0	$8,890	$0
Population-level STI costs	$71,010	$44,610	$28,600	$0
STI-attributable HIV costs	$34,120	$10,860	$74,010	$0
HIV costs averted through C&T	$0	$0	$0	$97,700
**Indirect costs averted**				
Indirect STI costs	$55,980	$31,640	$96,560	$0
Indirect HIV costs	$142,960	$45,490	$310,100	$409,390
**Total costs averted**	$389,950	$165,030	$575,360	$507,100

HIV C&T: HIV counseling and testing. Indirect HIV costs averted include the costs averted through prevention of STI-attributable HIV cases. Total costs may not match sum of direct and indirect costs due to rounding.

For the four hypothetical program activities in our example, the estimates of the averted costs ranged from $165,030 to $575,360. The highest estimate ($575,360) was obtained for the syphilis treatment scenario, despite the lower number of people treated (100) in this scenario. The estimate of the costs averted per person served in this scenario was notably higher than the other scenarios, and can be attributed to three main factors: the lifetime cost per case estimate we applied for syphilis was higher than that of gonorrhea or chlamydia, the benefits of preventing congenital syphilis were included, and, most importantly, the probability of an STI-attributable HIV infection was higher for syphilis than for gonorrhea and chlamydia.

## Discussion

The formulas developed in this study can be a useful tool to STI program personnel to generate evidence-based estimates of the economic impact of their program. The estimates generated by these formulas, when combined with estimates of program costs, can provide estimates of the net cost (program costs minus costs averted by program activities) of a program. Such estimates might be of value for those who want simply to compare the costs averted by their program to the overall budget of their program, as well as to those who want to develop estimates of the cost-effectiveness of their program activities. However, providing guidance for estimating the program costs of a specific STI prevention activity (such as STI screening in correctional settings) is beyond the scope of this manuscript.

The formulas we present are not reduced to more basic forms. For example, in the first formula in Table 4, the term "(0.16)(0.925)(0.70)($1,995)" is not reduced to "$207." We presented the formulas in this manner to facilitate adaptation of these formulas to non-US settings, or in US settings with substantially different input values, such as for the reduction in probability of PID associated with chlamydia treatment (0.16) or the direct medical costs associated with PID ($1,995). Presentation of the formulas in their longer forms allows for easier substitution of parameter values. We have developed a spreadsheet-based tool (available from the authors upon request) to facilitate the application of these formulas.

In the event that information on the sexual orientation of men served by a given program is unavailable or unreliable, estimates on the number of heterosexual men and MSM treated for each STI can be estimated based on the male-female ratio of STI cases in the population served by the program [88]. In a simplified application of the approach used by Heffelfinger and colleagues [88], the number of cases of a given STI in MSM can be estimated as the number of cases of that STI in men minus the number of cases of that STI in women (assuming there are more cases in men than in women).

In the event that information on the number of pregnant women treated for P&S syphilis is not known, this number can be estimated as the number of infected women treated for P&S syphilis (S_w_) multiplied by an adjustment factor to reflect the birth rate. For example, the adjustment term 0.066 could be applied in US settings, to reflect the birth rate among women ages 15 to 44 years in the US in 2004 (66 live births per 1,000 women) [89].

The formulas related to syphilis presented in Tables 1, 2, 3, 4, 5, 6 focus on P&S syphilis. The benefits of treatment of early latent syphilis cases could be included easily, by multiplying the number of early latent syphilis cases treated by the direct cost per case of syphilis ($572) and by the indirect cost per case of syphilis ($112). This adjustment conservatively assumes no benefit of treating early latent syphilis in terms of interrupting syphilis transmission, preventing congenital syphilis, and reducing HIV transmission.

### Key sources of uncertainty

There is uncertainty in the probability of PID in the absence of treatment for chlamydia and gonorrhea. We applied a 20% probability, which is in the lower portion of the often-cited range of 10% to 40% [5]. Nonetheless, it is possible that this 20% value overstates the probability of developing PID [90]. In light of the uncertainty in the probability of developing PID, we applied a cost per case of PID that falls in the lower end of the range of plausible values [23-25].

The average sequelae costs averted by syphilis treatment are not known with precision. To account for this uncertainty, we assumed that people with syphilis not treated by the STI program might seek treatment for syphilis elsewhere, or receive treatment inadvertently through antibiotics administered for an unrelated condition. This assumption reduced the expected sequelae costs of untreated syphilis by more than half, thereby making the estimates of the costs averted by syphilis treatment more conservative.

The formula for estimating the value of the interruption of STI transmission in the population applies an assumption that each case treated prevents 0.5 cases of that STI in the population. This assumption, though somewhat arbitrary, is likely conservative, because STI rates would decline if each new STI infection caused less than one more new infection [91]. In reality, reported rates of chlamydia, gonorrhea, and P&S syphilis in the US increased slightly in 2005 [92].

The formulas for estimating the reduction in STI-attributable HIV infections and for estimating the number of HIV infections averted by HIV counseling and testing are based on simple models, and may be more applicable for certain areas than others depending on factors such as HIV prevalence and HIV co-infection in people with STIs. However, the adjustments we applied (to account for partner overlap, for the impact of treatment on the interval in which an STI-attributable HIV infection might occur, and for the possibility that HIV cases averted are merely "delayed" rather than "forever averted") greatly reduced the estimated impact of program activities on HIV incidence. Of note, the probability of an STI-attributable HIV infection we applied for syphilis was substantially higher than that of gonorrhea or chlamydia. In the study from which these estimates were obtained, more conservative assumptions regarding the probability of HIV/STI coinfection were applied for gonorrhea and chlamydia than for syphilis, owing to the relatively plentiful studies of syphilis and HIV coinfection [70,93]. As such, the benefit of treating people with syphilis (relative to the benefit of treating people with gonorrhea or chlamydia) may be overestimated.

There is uncertainty in the indirect cost per case estimates, particularly in the instances when such estimates were not available from the literature and were calculated assuming an 0.5 ratio of indirect costs to direct medical costs per case (similar to that reported for the indirect cost of untreated chlamydia in females). The limited number of available estimates of indirect STI costs highlights the need for future studies in this area. Our estimates of the indirect costs included only lost productivity and excluded other indirect costs (such as foregone leisure time and time spent by family and friends for hospital visitations). Thus, the indirect cost estimates we applied may be conservative. For example, the indirect cost estimate we applied for congenital syphilis ($60,421) was substantially more conservative than that of a 1983 study which estimated the lifetime cost of special educational needs and reduced productivity per case of congenital syphilis at over $200,000 [94].

Clearly, estimating the economic impact of STI programs is an inexact exercise. However, to address the inherent uncertainty, we made numerous conservative assumptions as discussed above, such as applying a cost of PID in the lower range of plausible values, adjusting for partner overlap when estimating the impact of program activities on STI and HIV transmission, and assuming less impact of STI treatment on population-level STI incidence than would be expected given recent trends in reported STI rates in the US. The direct costs we applied for congenital syphilis and syphilis in adults were more conservative than the burden suggested in a cost-effectiveness study of a corrections-based syphilis screening program [95]. Furthermore, the calculations presented here may substantially understate the benefits of STI program activities because (1) not all of the potential benefits of treating people with chlamydia, gonorrhea, and P&S syphilis are included; (2) the benefits of preventing other STIs (such as genital herpes, human papillomavirus, hepatitis B, and trichomoniasis) are not included; and (3) we did not include intangible costs such as pain and suffering, which can be considerable.

### Resource allocation implications

The formulas presented here are intended to assist in the estimation of the economic impact of STI programs. Although the estimates resulting from these formulas can provide information relevant to resource allocation decisions, these formulas are not intended as a resource allocation tool, per se. First, our estimates of the costs averted by preventing a given STI may be overstated relative to the costs averted by preventing another STI. For example, as discussed above, the STI-attributable HIV costs for gonorrhea and chlamydia are more conservative than for syphilis. Second, our model is static and does not account for diminishing marginal returns. That is, in our model the benefits of STI treatment are constant regardless of the number of people treated. In reality, when prevention efforts are focused more intensely on one specific STI or on one specific population, the marginal benefits of prevention efforts would be expected to decrease (at some point). These limitations, as well as the uncertainties described above, should be considered when determining the utility of these formulas for informing resource allocation decisions.

## Conclusion

We provide a series of formulas that STI programs can use to generate estimates of the economic impact of their program activities, based primarily on published studies of the costs of STIs, the impact of STIs on HIV transmission, and the impact of HIV counseling and testing on HIV incidence. A spreadsheet-based tool to facilitate the application of these formulas is available from the authors upon request.

## Competing interests

The authors declare that they have no competing interests.

## Authors' contributions

DC and HWC originated the study. All authors participated in its design and in the development of assumptions on which the resulting formulas are based. HWC constructed the formulas and drafted the manuscript. All authors contributed to revisions of the manuscript, and read and approved the final manuscript.
